# A high-throughput screen of pharmacologically active compounds for inhibitors of UHRF1 reveals epigenetic activity of anthracycline derivative chemotherapeutic drugs

**DOI:** 10.18632/oncotarget.26889

**Published:** 2019-04-30

**Authors:** Hugh Giovinazzo, David Walker, Nicolas Wyhs, Jianyong Liu, David M. Esopi, Ajay M. Vaghasia, Yash Jain, Akshay Bhamidipati, Jianya Zhou, William G. Nelson, Srinivasan Yegnasubramanian

**Affiliations:** ^1^ Department of Pharmacology and Molecular Sciences, Johns Hopkins University School of Medicine, Baltimore, MD, USA; ^2^ Sidney Kimmel Comprehensive Cancer Center, Johns Hopkins University School of Medicine, Baltimore, MD, USA; ^3^ Department of Respiratory Disease, Thoracic Disease Center, The First Affiliated Hospital College of Medicine, Zhejiang University, Hangzhou, China PR; ^4^ Current Affiliation: Department of Pediatric Oncology, Dana Farber Cancer Institute, Harvard Medical School, Boston, MA, USA; ^5^ Current Affiliation: Active Motif, Carlsbad, CA, USA

**Keywords:** DNA methylation, UHRF1 inhibitors, SRA domain, TR-FRET, anthracyclines

## Abstract

DNA methylation can mediate epigenetic silencing of tumor suppressor and cancer protective genes. The protein ubiquitin-like containing PHD and ring finger domains 1 (UHRF1) is an essential component in cells for DNA methylation maintenance. The SET- and RING-associated (SRA) domain of UHRF1 can bind hemimethylated DNA, and mediate recruitment of DNA methyltransferases to copy the methylation pattern to the newly synthesized daughter strand. Loss of UHRF1 function can lead to demethylation and re-expression of epigenetically silenced tumor suppressor genes and can reduce cancer cell growth and survival. We created a high-throughput time-resolved fluorescence resonance energy transfer (TR-FRET) assay to screen for inhibitors capable of disrupting the interaction between the UHRF1-SRA domain and hemimethylated DNA. Using this assay (Z’ factor of 0.74 in 384-well format) we screened the Library of Pharmacologically Active Compounds (LOPAC) for UHRF1-SRA inhibitors, and validated 7 hit compounds. These compounds included the anthracycline derivatives idarubicin and mitoxantrone, which are commonly used chemotherapeutic drugs known to mediate cytotoxicity by acting as class II topoisomerase (TOP2) poisons. In a panel of additional known topoisomerase poisons, only the anthracycline derivatives showed dose responsive inhibition of UHRF1-SRA. Additionally, mitoxantrone and doxorubicin showed dose-responsive global DNA demethylation and demonstrated a synergistic growth inhibition of multiple cancer cell lines when combined with the DNA methyltransferase (DNMT) inhibitor decitabine. These data validate a novel TR-FRET assay for identification of UHRF1 inhibitors, and revealed unexpected epigenetic properties of commonly used chemotherapeutic drugs that showed synergistic cytotoxicity of cancer cells when combined with a demethylating agent.

## INTRODUCTION

Cytosine methylation at cytosine-phospho-guanine (CpG) dinucleotides is a common epigenetic alteration in virtually all human somatic tissues. The presence of a methyl group on the 5 position of a cytosine base in DNA can serve as a signal for repressing gene expression, especially when found in gene regulatory regions. Aberrations in DNA methylation patterns are found in nearly all human cancers; hypermethylation of gene promoters has been causally implicated in epigenetic repression of key tumor suppressor genes [1]. These hypermethylation events can be stably maintained, and be subject to clonal selection during cancer progression [2, 3]. Consequently, pharmacologic reversal of these DNA hypermethylation events, via inhibition of the molecular machinery responsible for establishing and maintaining them, is an attractive avenue for cancer therapy.

Currently the only FDA-approved drugs targeting the DNA methylation machinery are the nucleoside analogs azacitidine and decitabine. Both drugs inhibit DNA methyltransferases through incorporation into genomic DNA in lieu of a native cytosine, leading to covalent trapping and degradation of DNMTs [4]. However, these drugs exhibit many of the toxicities of other nucleoside analog drugs, including anemia, thrombocytopenia and neutropenia [5]. Ideally, a non-nucleoside inhibitor of the methylation machinery would achieve the same therapeutic effect but with fewer side effects, and for this reason, multiple efforts for developing non-nucleoside DNMT inhibitors are currently underway [6–10].

Another interesting target for development of non-nucleoside inhibitors of DNA methylation maintenance is the protein ubiquitin-like containing PHD and RING finger domains 1 (UHRF1), which has been implicated as a critical component of the DNA methylation maintenance machinery in actively dividing cells [11–16], reviewed in [17]. In S-phase, UHRF1 binds newly replicated DNA in its hemimethylated state through its SRA domain and recruits DNA methyltransferase 1 (DNMT1) to copy the methylation code to the daughter DNA strand in a manner analogous to semi-conservative replication [11, 12, 18]. The SRA domain plays the central role in UHRF1 function [19, 20], including mediating interactions with hemi-methylated DNA that allosterically modulate interactions with other UHRF1 domains and their histone substrates [21]; its SRA domain is therefore a key target for development of UHRF1 inhibitors.

UHRF1 is overexpressed in a number of cancer types, and its depletion has been shown to inhibit growth and invasion of cancer cells [18, 20]. Despite such credentialing of UHRF1 as a target for cancer therapy, to date there have been no published attempts to our knowledge to identify small molecule UHRF1 inhibitors, in part due to a lack of robust assays for identification of such inhibitors.

We have developed a robust time-resolved fluorescence resonance energy transfer (TR-FRET) assay to identify compounds that inhibit the binding of the SRA domain of UHRF1 to hemimethylated DNA. The assay was adapted to 384-well plate format and was tested on a pilot screen of 1,280 pharmacologically active compounds, successfully identifying hits capable of inhibiting the interaction of the UHRF1-SRA domain with hemimethylated DNA. These screening efforts led to identification of unexpected epigenetic properties of multiple anthracycline derivative chemotherapeutic drugs, warranting further testing of combinations of these drugs with existing DNA demethylating agents for enhanced cancer control.

## RESULTS

### Measurement of UHRF1_SRA domain binding to DNA using TR-FRET

**Figure 1:**
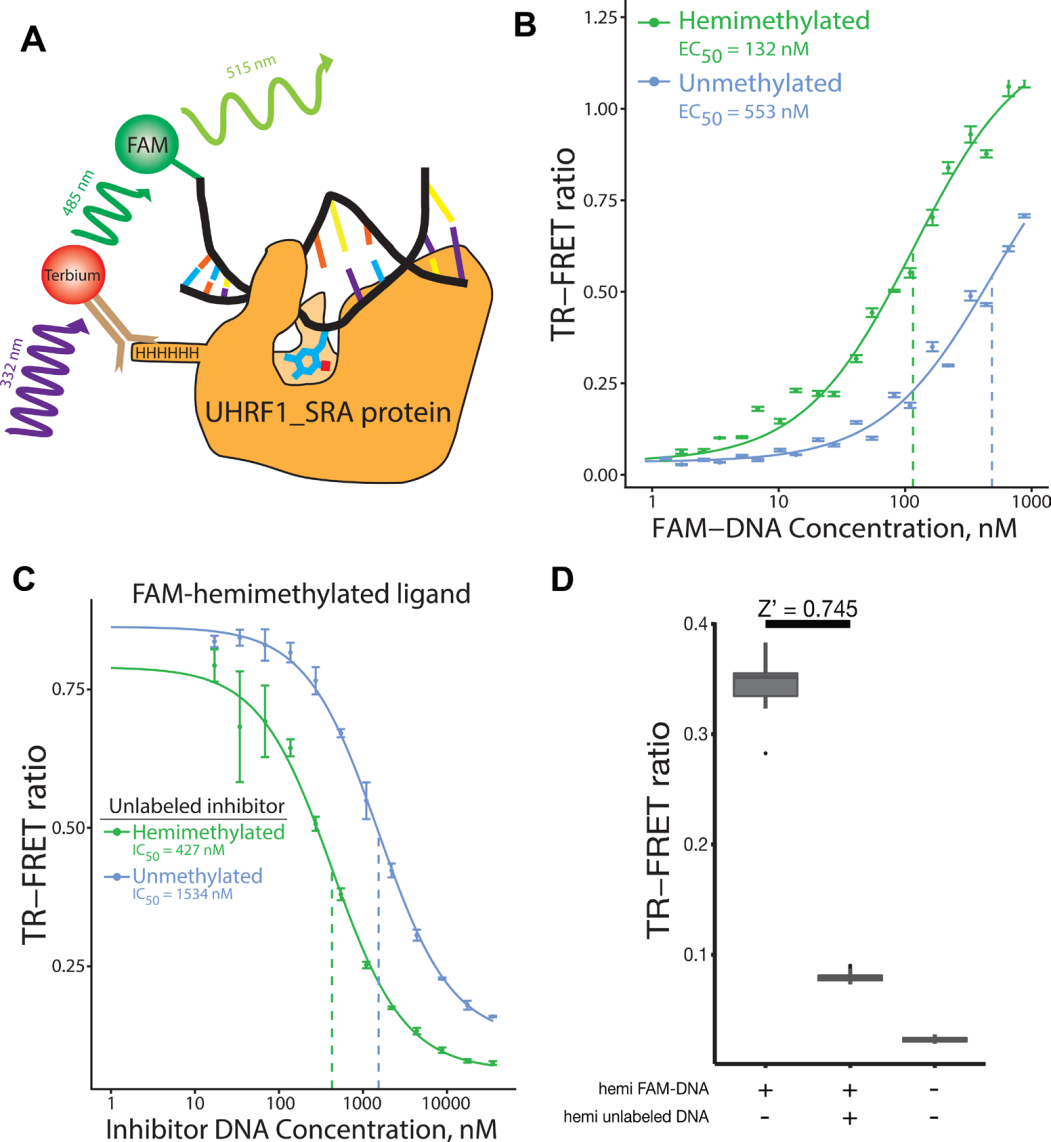
Overview, validation, and evaluation of performance of TR-FRET UHRF1_SRA DNA binding assay. (**A**) Schematic of the TR-FRET assay: UHRF1_SRA protein complexes with FAM-labled, hemimethylated DNA and a terbium-conjugated antibody that interacts with the 6xHis tag on UHRF1_SRA. A FRET pair is formed upon binding; the ratio of 488 nm to 515 nm emission is a measure of protein binding to DNA. (**B**) Binding curve of UHRF1_SRA to hemi- and unmethylated DNA. Hemimethylated ligand binding occurs with an estimated EC_50_ value of 132 nM. Unmethylated DNA shows significantly lower binding with an estimated EC_50_ of 553 nM, although this may be underestimated since the binding did not reach saturation at the DNA concentrations tested. (**C**) Binding competition assay using an unlabeled hemimethylated or unmethylated oligos. As expected, the unlabeled hemimethylated oligo showed more potent inhibition than the unlabeled unmethylated oligo. (**D**) 32 replicates of three conditions were performed in 384 well plate format: i) UHRF1_SRA domain (125 nM) with FAM-labeled hemimethylated DNA (100 nM) without any inhibitor, ii) same as (i) but with addition of an excess of “perfectly competitive” hemimethylated unlabeled DNA inhibitor (5 μM), and iii) UHRF1_SRA domain polypeptide alone without any DNA ligand or inhibitor. The calculated Z’ factor between conditions (i) and (ii) is 0.745. For (B and C), points represent mean TR-FRET ratio, error bars represent standard error, and EC_50_ values are indicated with dotted vertical lines.

We developed a TR-FRET assay to measure binding of the SRA domain of UHRF1 to various DNA substrates (Figure 1A). Using this assay, we measured the affinity of the UHRF1_SRA domain for hemimethylated and unmethylated double strand DNA oligonucleotides (Figure 1B). The UHRF1_SRA protein bound hemimethylated DNA preferentially compared to the unmethylated oligonucleotides (EC_50_ of 132 nM vs 553 nM, respectively) (Figure 1B). To confirm this finding, we tested the ability of unlabeled hemimethylated or unmethylated DNA to inhibit UHRF1_SRA binding to a labeled hemi-methylated oligo. The perfect competitive unlabeled hemi-methylated DNA inhibited binding of the labeled hemi-methylated ligand with an IC_50_ of 427 nM, while the unlabeled unmethylated DNA inhibited the labeled hemi-methylated DNA with an IC50 of 1.53 μM (Figure 1C). This assay therefore confirmed the preferential binding of UHRF1-SRA to hemimethylated DNA compared to unmethylated DNA, and was able to detect inhibition of binding using a perfect-competitive inhibitor (unlabled hemi-methylated DNA).

Using the above experiments, we optimized the concentrations, buffer conditions and incubation time of the assay for 384 well plate format. To test its performance, we evaluated assay repeatability using three control reactions at 32 replicates each (Figure 1D): i) no inhibitor control; ii) excess “perfectly competitive inhibitor” (unlabeled hemimethylated DNA) control; and iii) a technical control with no DNA present. As expected, unlabeled hemimethylated DNA greatly decreased the signal compared to the no inhibitor control. The calculated Z’ factor between the negative (i) and positive (ii) inhibitor controls was 0.745, indicating that the assay had robust signal-to-noise ratios in 384 well plate format to facilitate high-throughput screening experiments.

### Screen of LOPAC for UHRF1_SRA inhibitors

**Figure 2:**
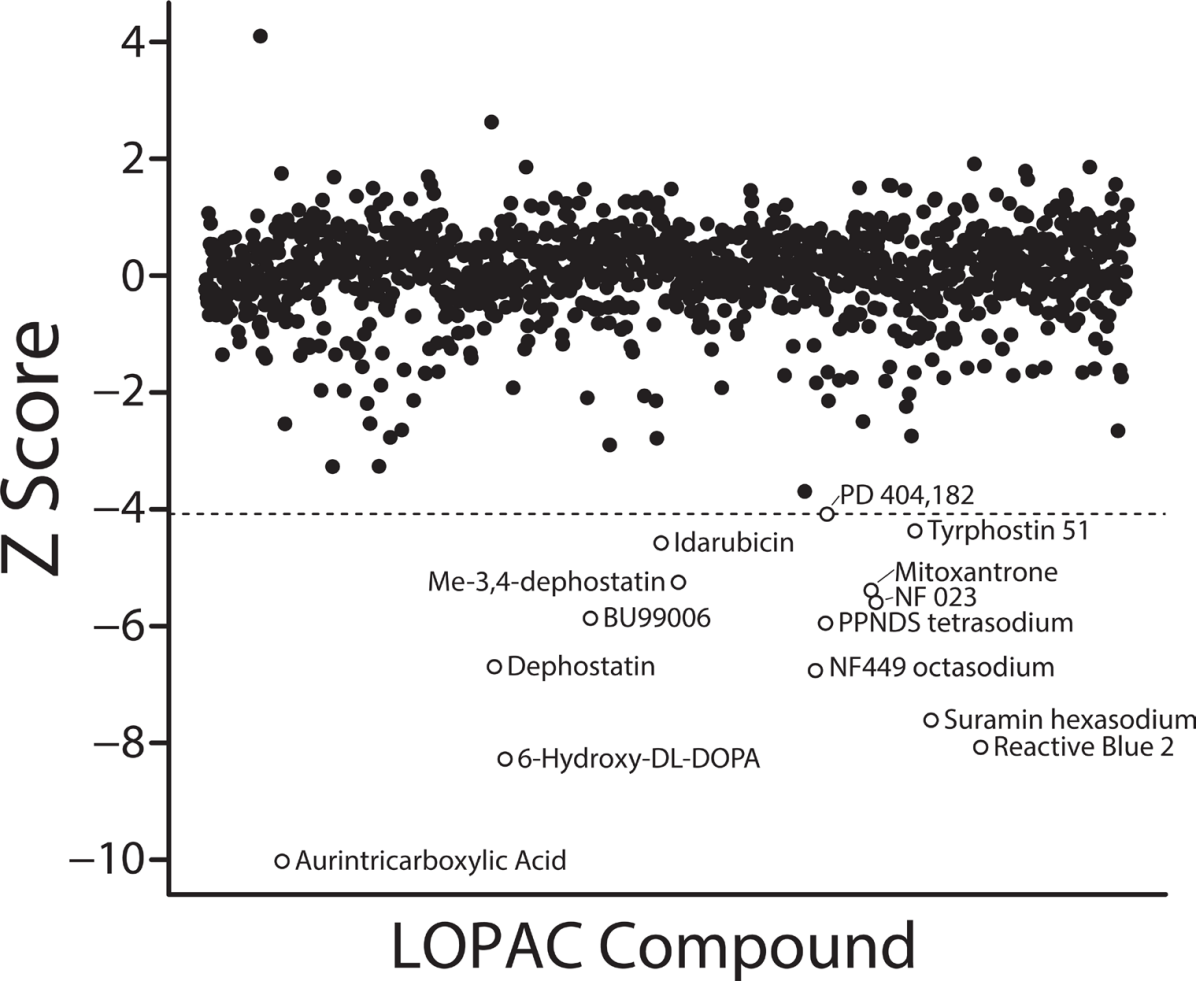
Results of LOPAC library screen. 1280 compounds were screened for inhibitor activity against UHRF1_SRA binding to hemimethylated DNA. TR-FRET ratios from each compound were normalized to the plate median and standard deviation to calculate a standardized Z score as discussed in the Materials and Methods. The Z score corresponding to a Bonferroni-adjusted *p* value of 0.05 is labeled as the dotted line, and compounds falling below this threshold were considered hits, labeled as open circles.

Using our TR-FRET assay, we next carried out a pilot high-throughput screen for UHRF1_SRA domain inhibitors among 1280 compounds assembled in the Library of Pharmacologically Active Compounds (LOPAC), which contains a diverse set of chemicals with known pharmacological targets and functions. Compounds were evaluated at a concentration of 20 μM. While the majority of the library did not inhibit UHRF1_SRA domain binding to hemimethylated DNA, 14 active compounds exhibited significant inhibition (Figure 2, open circles; Bonferroni adjusted *p*-value < 0.05).

### Validating inhibitors of UHRF1_SRA binding to hemimethylated DNA

**Figure 3:**
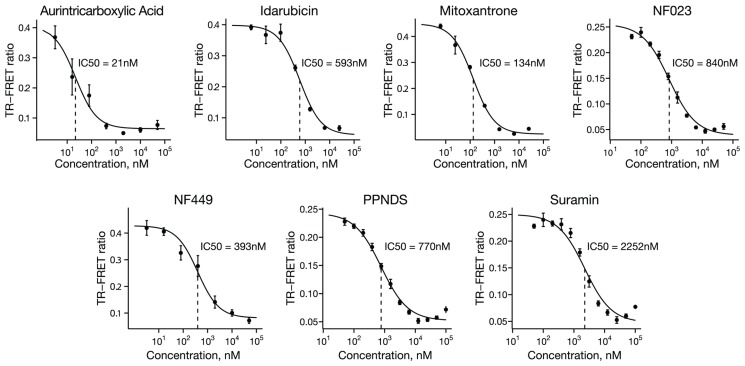
Dose-response confirmation of screen hits. Seven of the hit compounds produced sigmoidal dose-response curves, providing validation of the primary screen and also a measure of the potency of inhibition. Points represent mean TR-FRET ratio, error bars represent standard error, and IC_50_ values are indicated with dotted vertical lines.

Compounds that significantly inhibited the binding of UHRF1_SRA protein to hemimethylated DNA in the pilot screen were evaluated in a dose-response fashion as validation and to assess potency. One compound, Reactive Blue 2, was omitted from dose response testing because its coloration and light absorption could optically interfere with the assay. Of the remaining 13 compounds, 7 inhibited binding with a sigmoidal dose response (Figure 3; aurintricarboxylic acid, idarubicin, mitoxantrone, NF023, NF449, PPNDS and Suramin), while 6 failed to show any appreciable inhibition (6-OH-DL-DOPA, PD404,182, tyrphostin 51, methyl-3,4-dephostatin, BU99006, and dephostatin).

### Anthracycline derivative chemotherapeutic drugs inhibit UHRF1-SRA binding to hemimethylated DNA and synergize with known DNA methyltransferase inhibitors for cancer cell cytotoxicity

Two of the validated inhibitors, idarubicin and mitoxantrone, are anthracycline derivatives widely used for cancer treatment and are thought to exert their major cytotoxic effects via inhibition of type II topoisomerases (TOP2). To understand whether this was a property of anthracycline derivative compounds or of topoisomerase inhibitors more generally, we tested a panel of other known anthracycline derivative chemotherapeutic drugs (daunorubicin, doxorubicin, pixantrone), and other topoisomerase inhibitors (TOP2 inhibitors merbarone and etoposide, and TOP1 inhibitors topotecan and S-camptothecin). The anthracycline derivatives have a similar mechanism of action mediating cytotoxicity that involves DNA intercalation, stabilization of TOP2-DNA cleavage complexes and generation of DNA double strand breaks [22]. Merbarone is a catalytic inhibitor of TOP2 enzymes that prevents formation of the cleavage complex [23]. Etoposide does not intercalate with DNA alone, but rather binds and stabilizes the TOP2-DNA cleavage complex ultimately leading to generation of a double strand break [24]. Finally, topotecan and S-camptothecin act as type I topoisomerase (TOP1) poisons [25].

**Figure 4:**
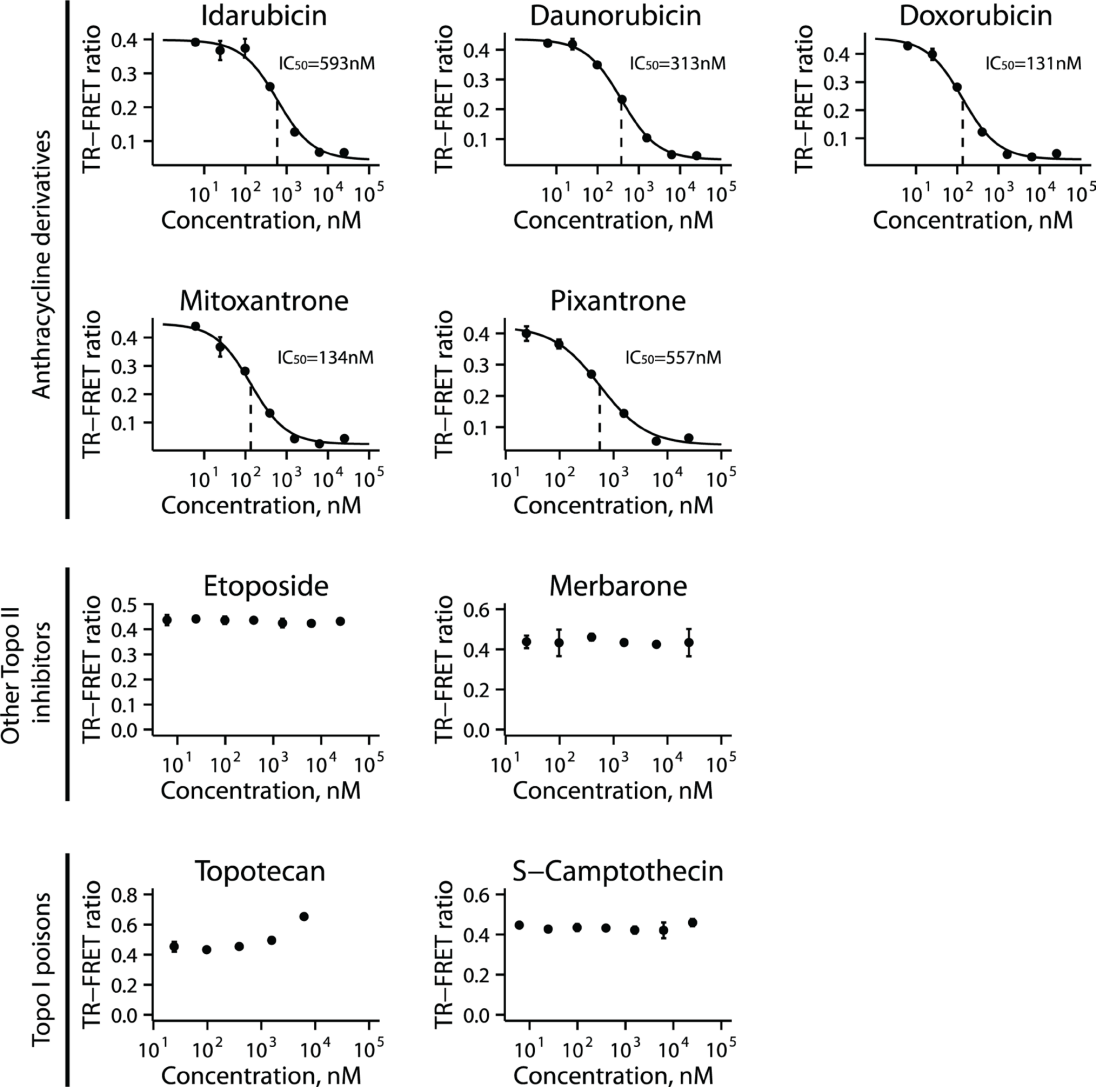
Activity of topoisomerase inhibitors for UHRF1_SRA inhibition. A collection of anthracycline derivatives, other topoisomerase II inhibitors, and topoisomerase I inhibitors were tested in a dose-response fashion in the UHRF1_SRA TR-FRET assay. Points represent mean TR-FRET ratio, error bars represent standard error, and IC_50_ values are indicated with dotted vertical lines.

We tested each compound in a dose-response on the UHRF1_SRA TR-FRET assay. From the three classes of inhibitors (anthracycline derivatives, non-anthracycline TOP2 inhibitors, and TOP1 inhibitors) only the anthracycline derivatives showed inhibition, with IC_50_ values ranging from 131 to 593 nM (Figure 4).

**Figure 5:**
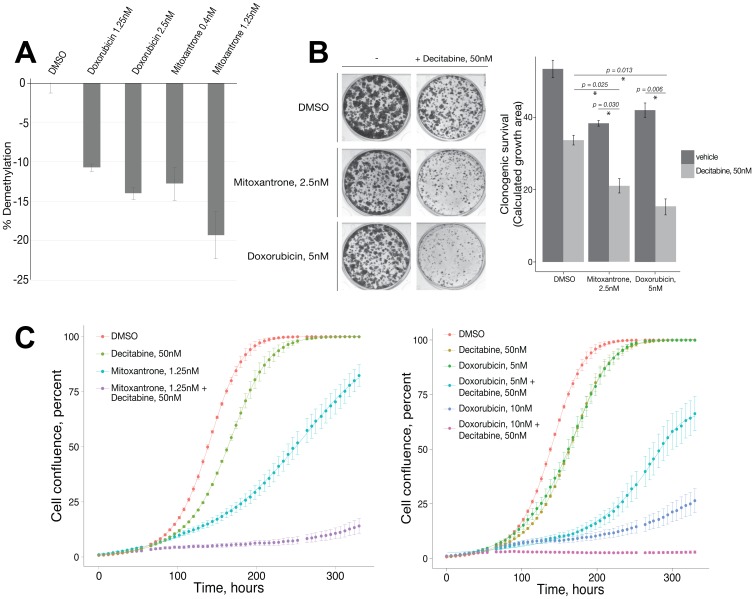
Mitoxantrone and doxorubicin treatment causes genomic hypomethylation and synergizes with decitabine for inhibition of cancer cell growth/survival. (**A**) Percent reduction in methylation of genomic DNA of DU-145 cells upon treatment with Doxorubicin (1.25 nM and 2.5 nM) and Mitoxantrone (0.4 nM and 1.25 nM) measured using LC/MS/MS. (**B**) Clonogenic survival of DU-145 cells given compounds at indicated doses alone and in combination with 50 nM decitabine. Bar graph represents calculated mean cumulative colony surface area of duplicate treatments. (**C**) Cellular growth/proliferation curves. Combinations of mitoxantrone or doxorubicin at indicated doses with 50 nM decitabine were added to DU-145 cells, with DMSO, decitabine, mitoxantrone, or doxorubicin alone as controls. For all graphs, error bars represent standard deviation. ^*^ indicates *p* < 0.05.

Since UHRF1 and DNMT1 cooperate to maintain DNA methylation [11, 12], we reasoned that the anthracycline derivative UHRF1 inhibitors identified here should induce DNA demethylation, and could have synergistic cancer cytotoxic activity with known DNMT inhibitors such as decitabine. Initially, we evaluated the ability for doxorubicin and mitoxantrone to reduce genomic methylation in DU-145. After five days of exposure to doxorubicin (1.25 nM and 2.5 nM) and mitoxantrone (0.4 nM and 1.25 nM) a demethylation of 10%–20% was measured relative to vehicle control DMSO (Figure 5A). We then exposed DU145 cells to vehicle control, decitabine alone, mitoxantrone or doxorubicin alone, or mitoxantrone or doxorubicin in combination with decitabine, and assessed clonogenic survival. Interestingly, at doses in which decitabine, mitoxantrone, and doxorubicin treatment alone exhibited some reduction in clonogenic survival, the combination of decitabine with either mitoxantrone or doxorubicin resulted in significantly enhanced reduction in clonogenic survival (Figure 5B). Growth curve analyses provided further confirmation of the synergy between decitabine and mitoxantrone or doxorubicin (Figure 5C), a finding that was robust in multiple cancer cell line models (Supplementary Figure 1). Taken together, these findings suggest that commonly used anthracycline chemotherapeutic drugs, including mitoxantrone and doxorubicin, can synergize with known DNMT inhibitors for cancer cell cytotoxicity.

## DISCUSSION

Recent studies have shown that UHRF1 is a critical epigenetic mediator that can bind newly replicated, hemimethylated DNA and recruit DNMT enzymes to facilitate maintenance of DNA methylation after DNA replication [11, 12, 16]. UHRF1 has been credentialed as a target for cancer therapeutics [18, 20]; development of inhibitors of UHRF1 binding to hemimethylated DNA could yield a novel class of DNA demethylating agents that can relieve the DNA hypermethylation mediated epigenetic repression of tumor suppressor genes in cancer cells while potentially avoiding the pitfalls and toxicities of existing nucleoside analog DNMT inhibitor DNA demethylating agents [20, 26]. We have developed and optimized a TR-FRET assay to screen compound libraries for inhibitors capable of disrupting the binding of the SRA domain of UHRF1 to hemimethylated DNA. The assay allowed quantitative analysis of UHRF1_SRA binding to hemimethylated DNA and showed strong signal to noise ratios amenable to high-throughput screening.

Having established the performance of the TR-FRET binding assay, we used it to screen a pilot library of 1,280 pharmacologically active compounds and identified multiple hits capable of inhibiting UHRF1_SRA binding to hemimethylated DNA. In future studies, this assay can be used to screen much larger and diverse compound libraries to identify selective inhibitors of UHRF1_SRA binding to hemimethylated DNA.

Two of the confirmed hits, mitoxantrone and idarubicin, are anthracycline derivatives known to inhibit type II topoisomerases via DNA intercalation [27, 28]. Among a panel of other anthracycline derivatives and other topoisomerase inhibitors, we saw that only the anthracycline derivative compounds, including doxorubicin, daunorubicin, idarubicin, pixantrone and mitoxantrone, were capable of inhibiting UHRF1 binding to hemimethylated DNA. We found that these compounds could result in genomic demethylation and synergize with known DNA methyltransferase inhibitors for inhibition of clonogenic survival and cancer cell growth. Whether this synergistic cytotoxicity is mediated through combined targeting of DNMT and UHRF1 is still not known and can be evaluated in future studies. Based on these data, we can speculate that combination of these anthracycline derivative chemotherapeutic drugs with DNA methyltransferase inhibitors or other epigenetic drugs may result in enhanced therapeutic efficacy, a notion that suggests further preclinical and clinical testing. In support of this, a recent phase I clinical trial for treatment of diffuse large B-cell lymphoma demonstrated high rates of complete clinical response of a doxorubicin containing chemotherapeutic regimen when given after a period of treatment with a DNA methyltransferase inhibitor [29].

In summary, we have developed a novel TR-FRET high-throughput screening assay for identification of compounds capable of inhibiting the interaction of UHRF1-SRA to hemimethylated DNA. Application of this assay in a pilot screen of pharmacologically active compounds and follow up studies revealed unexpected epigenetic activity of commonly used anthracycline derivative chemotherapeutic drugs, a property that might be exploitable for combination therapy with existing epigenetic drugs including DNA methyltransferase inhibitors and HDAC inhibitors. Further studies with our TR-FRET assay on large chemical compound libraries may yield novel UHRF1 inhibitors, representing a novel class of DNA demethylating agents for development as pharmacological probes of epigenetic mechanisms and as drugs for cancer therapy.

## MATERIALS AND METHODS

### Construction of oligonucleotides

Hemimethylated and unmethylated double-stranded DNA 16-mers were generated by annealing complimentary oligonucleotides: top 5′-(FAM)-CATGCT XGTAGCAGAG-3′ and bottom 5′- CTCTGCTAXGA GCATG-3′, where FAM is a fluorescein label and X is a cytosine that is unmethylated or methylated at the 5-position (Integrated DNA Technologies, Coralville, Iowa, USA). Combinations of top and bottom sequences were annealed by heating the oligonucleotides to 95° C and cooling at 2° C per minute down to a temperature of 10° C to generate hemi-methylated and unmethylated duplex DNA oligos.

### Production of UHRF1_SRA protein

A codon-optimized sequence of the SRA domain of UHRF1 was synthesized in a pUC19 plasmid (GenScript USA Inc., Piscataway, NJ, USA), and cloned into the HIS-tag containing vector pEXP-CT (Life Technologies, Grand Island, NY, USA). This vector was then transformed into BL-21 Gold cells (Agilent Technologies, Inc., Santa Clara, CA, USA) and cultured at 37° C at 220 rpm in an orbital shaker to log phase (OD_600_ of 1.0) in Terrific Broth media (Corning Cellgro, Corning, NY, USA). Cultures were then induced for protein expression using 1 mM isopropyl β-D-1-thiogalactopyranoside (IPTG, Corning Cellgro, Corning, NY, USA) at 24° C overnight. Bacterial cultures were harvested by centrifugation at 3,000 x *g* for 15 minutes at 4° C. Isolated bacterial cells were resuspended in an equilibrium buffer consisting of 300 mM NaCl, 50 mM sodium phosphate pH 8.0, 5 mM imidazole (Sigma-Aldrich, St. Louis, MO, USA), and a protease inhibitor cocktail (Roche Diagnostics GmbH, Mannheim, Germany). Lysis was performed by adding 0.75mg/mL lysozyme followed by incubation for 30 minutes at room temperature. Additionally, the bacterial mixture was sonicated three times (Sonic Dismembrator Model 100, Thermo Fisher Scientific, Waltham, MA) for 10 seconds followed by 30 seconds on ice. The mixture was centrifuged at 10,000 × *g* for 30 minutes to remove non-soluble components. IMAC Nickel NTA beads (Bio-Rad, Hercules, CA, USA) were prepared by washing with deionized water followed by equilibrium buffer. The bacterial supernatant was mixed with washed beads and incubated at 4° C for 1 hour. The beads were collected on a filter column and washed with 5 solutions of increasing imidazole concentration (15, 20, 25, 30, 45 mM). The beads were then eluted in equilibrium buffer containing 250 mM imidazole and incubated at 4° C for 20 minutes. The eluate was collected by gravity flow and dialyzed into a protein storage buffer containing 125 mM NaCl, 20 mM Tris pH 7.4, and 10 mM 2-mercapto ethanol using the Slide-A-Lyzer system (Pierce, Rockford, IL, USA).

### TR-FRET assay for UHRF1_SRA binding to methylated DNA

TR-FRET assays were performed in an optimized TR-FRET buffer (4% glycerol, 1 mM MgCl_2_, 0.5 mM EDTA, 0.5 mM DTT, 125 mM NaCl, 10 mM Tris HCl, and 0.2% Tween-20) in non-binding, flat-bottom 384-well plates (Corning) [30]. UHRF1_SRA protein was added to a final concentration of 125 nM and terbium-labeled antibody was added at 5 nM (Life Technologies) in a final reaction volume of 20 μL. For initial binding experiments examining the affinity of UHRF1_SRA to DNA, FAM-labeled hemimethylated or unmethylated double stranded oligonucleotides were added in a dilution series, and the reaction mixture was incubated at 4°C with shaking for 1 hour. TR-FRET measurements were made on a SpectraMax M5 (Molecular Devices, Sunnyvale, CA, USA), using an excitation wavelength of 332 nm and emissions read at 485 nm and 515 nm with a 50 μsec delay and 400 μsec integration. The ratio of the 515 nm emission (arising from FRET) to the 485 nm emission provides a normalized TR-FRET signal that represents the extent of binding. For inhibitor curves, FAM-labeled hemimethylated oligonucleotide was added to a final concentration of 100 nM, and unlabeled oligonucleotide was added in a dilution series. A Z’ factor was calculated for 32 replicates of UHRF1_SRA protein binding FAM-labeled hemimethylated DNA with or without the addition of an excess (5 μM) of unlabeled hemimethylated inhibitor (“cold” perfect competitive inhibitor positive control for inhibition):

$$Z^{'}=1-3\left(\frac{SD_{h}-SD_{i}}{\left|{\text{µ}}_{h}-{\text{µ}}_{i}\right|}\right)$$

where SD_h_ and *μ*_h_ refer to the standard deviation and mean of binding to the FAM-labeled hemimethylated substrate without the perfect competitive inhibitor, and SD_i_ and *μ*_i_ refer to the standard deviation and mean of binding the FAM-labeled hemimethylated substrate in the presence of excess unlabeled hemimethylated perfect competitive inhibitor.

### LOPAC1280TM screen

The above TR-FRET assay was employed to screen the Library of Pharmacologically Active Compounds (LOPAC) (Sigma-Aldrich) using optimized conditions. 200 uM stock solutions of compounds dissolved in dimethyl sulfoxide (DMSO) were added to a final concentration of 20 μM across four 384-well plates containing 20 μL of TR-FRET buffer with 125 nM UHRF1_SRA protein, 100 nM double stranded FAM-labeled hemimethylated oligos, and 5 nM terbium labeled anti-HIS antibody. Controls on each plate include: 1) a DMSO-only vehicle control, 2) 5μM of unlabeled, methylated oligonucleotide as a positive inhibition control, 3) unmethylated, FAM-labeled oligonucleotide as a negative control, and 4) no UHRF1_SRA protein negative control. Each plate was incubated at 4° C for one hour and read using a Safire^2^ (Tecan Group, Männedorf, Switzerland) with excitation at 332 nm and emissions read at 485 nm and 515 nm. A Z score of the TR-FRET signal (ratio of 515 nm signal to the 485 nm signal) was calculated for each compound using the following formula:

$$\;Z\;score=\frac{x-\bar{x}}{\sigma }$$

where *x* is the TR-FRET signal for each compound, $\bar{x}$ is the median TR-FRET signal of all compounds on the plate, and *σ* is the standard deviation of all compound TR-FRET signals across the plate. Hits were defined as those compounds with Z-score < −4.08 corresponding to a Bonferroni adjusted *p*-value of less than 0.05.

### Secondary validation of LOPAC hits

Each hit compound was tested using the TR-FRET assay above in quadruplicate in a dilution series (50 μM to 0.64 nM). Curve fitting was performed using the open source software R version 2.15.2 with the ggplot2 package.

### DNA methylation liquid chromatography-mass spectrometry assay

5-methyl-2’-deoxycytidine genomic content was measured using LC-MS as described previously [31]. Briefly, genomic DNA was extracted and digested into single nucleosides. Chromatographic separation was achieved with a Thermo Hypercarb porous graphite column (100 mm × 2.1 mm, 5 μm) and isocratic elution with a 10 mM ammonium acetate:acetonitrile with 0.1% formic acid (70:30, v/v) mobile phase over a 5 min total analytical run time. An AB Sciex 5500 triple quadrupole mass spectrometer operated in positive electrospray ionization mode was used for the detection of 5-methyl-2′-deoxycytidine.

### Growth curve assay

Five hundred DU-145 cells were seeded per well in 48 well plates. The next day, cells were treated with a single dose of DMSO (vehicle control), decitabine alone (50 nM), mitoxantrone alone (1.25 nM), doxorubicin alone (5 nM or 10 nM), or decitabine in combination with mitoxantrone (1.25 nM) or doxorubicin (5 nM or 10 nM). Cell growth was measured using IncuCyte™ technology (Essen Bioscience, Ann Arbor, MI, USA) by imaging 16 fields per well at 6 hour intervals. The cell surface area and percent confluence were calculated using IncuCyte software, and growth curves were constructed in R using the ggplot2 package.

### Clonogenic survival assay

Two thousand DU-145 cells were seeded per well in 6 well plates. The next day, decitabine (Sigma-Aldrich) was freshly dissolved in DMSO to make a 10 mM stock solution, and diluted with media to a concentration of 5 μM, and was then added into the culture plates to a final concentration of 50 nM within 15 minutes. Mitoxantrone and doxorubicin (Sigma-Aldrich) were dissolved in DMSO to make a 10 mM stock, and similarly diluted in media to a concentration of 5 μM before being added to the culture plates for final concentrations of 2.5 nM for mitroxantrone and 5 nM for doxorubicin. Colony growth was confirmed by light microscopy 10 days later, and cells were stained with crystal violet (0.5% crystal violet, 25% methanol in PBS) for 30 minutes, washed twice with running water, and air-dried. The total growth area of all colonies was measured using a G-BOX imager (Syngene USA, Frederick, MD, USA).

## SUPPLEMENTARY MATERIALS


