# Supplementary material for: A high-throughput screen of pharmacologically active compounds for inhibitors of UHRF1 reveals epigenetic activity of anthracycline derivative chemotherapeutic drugs
Source: Oncotarget. 2019 Apr 30;10(32):3040–50. doi: 10.18632/oncotarget.26889 (PMC6508961; doi:10.18632/oncotarget.26889)
Supplement: Supplementary file 1 [file oncotarget-10-3040-s001.pdf]

A high-throughput screen of pharmacologically active compounds for inhibitors of UHRF1 reveals epigenetic activity of anthracycline derivative chemotherapeutic drugs

SUPPLEMENTARY MATERIALS

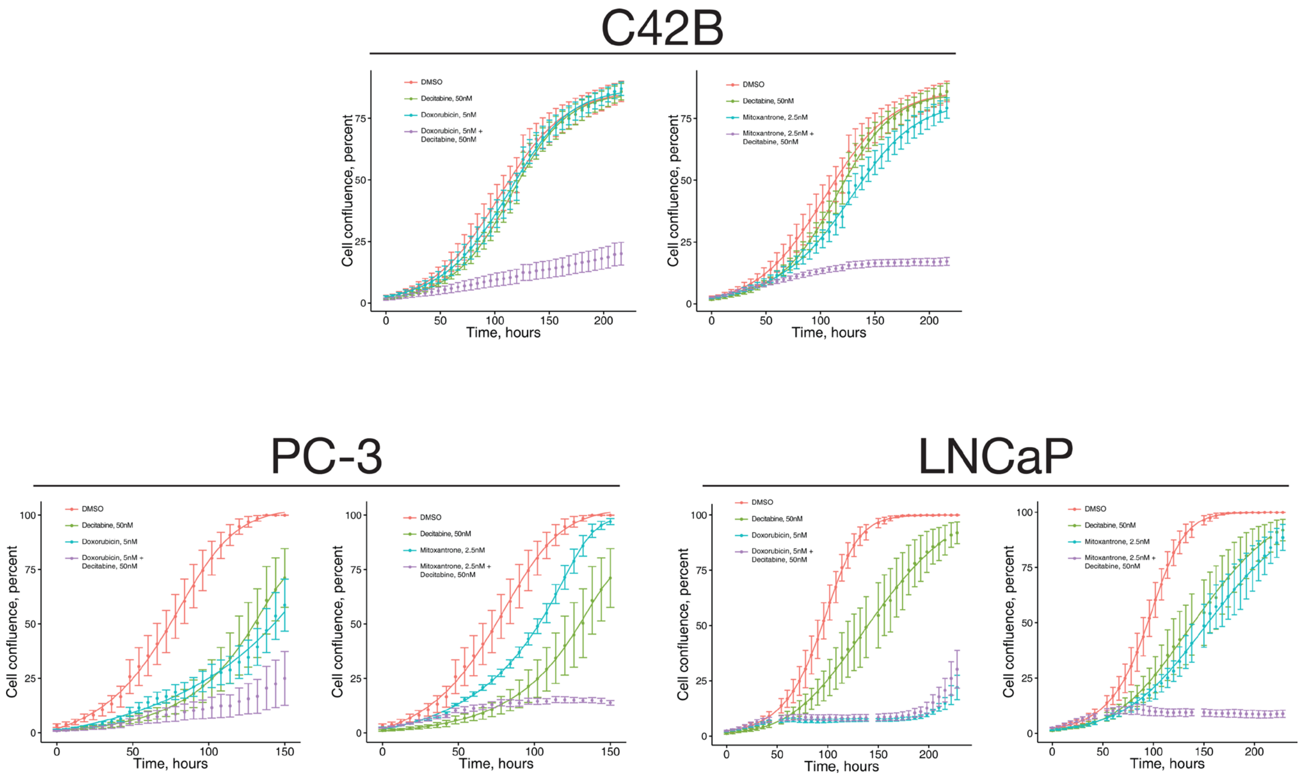

Supplementary Figure 1: Cellular growth/proliferation assays of C42B, PC-3 and LNCaP prostate cancer cell lines treated with mitoxantrone (2.5 nM) or doxorubicin (5 nM) in combination with decitabine (50 nM) or vehicle control (DMSO), decitabine alone, or DMSO alone. The combination of mitoxantrone with decitabine showed enhanced inhibition of growth/proliferation compared to either drug alone in all three cell lines. The combination of doxorubicin and decitabine showed enhanced inhibition of growth/proliferation compared to either drug alone in C42B and PC-3 cells, but did not show enhanced growth inhibition compared to doxorubicin alone at the selected dose in LNCaP cells.
